# Feeding Challenges in Trisomy 21: Prevalence and Characteristics of Feeding Disorders and Food Neophobia—A Cross-Sectional Study of Polish Children and Adolescents with Down Syndrome

**DOI:** 10.3390/nu17122030

**Published:** 2025-06-18

**Authors:** Agnieszka Białek-Dratwa, Sebastian Żur, Adam Sokal, Wiktoria Staśkiewicz-Bartecka, Oskar Kowalski

**Affiliations:** 1 Department of Human Nutrition, Department of Dietetics, Faculty of Public Health in Bytom, Medical University of Silesia in Katowice, Jordana 19, 41-808 Zabrze, Poland; 2 Department of Food Technology and Quality Evaluation, Department of Dietetics, Faculty of Public Health in Bytom, Medical University of Silesia in Katowice, Jordana 19, 41-808 Zabrze, Poland; wstaskiewicz@sum.edu.pl

**Keywords:** children, Down’s syndrome, food neophobia, feeding disorders

## Abstract

Background: Food neophobia, defined as reluctance to try new foods, may lead to nutritional deficiencies and complicate dietary management—especially in individuals with Down syndrome, who often present with oral-motor dysfunction. This condition may result in nutritional deficiencies and difficulties in adhering to dietary recommendations, particularly in individuals with comorbidities. In individuals with Down syndrome (DS), who frequently present with oral motor disorders and chronic diseases, the problem may be especially pronounced. Objectives: The aim of the study was to assess the risk of food neophobia and feeding difficulties in children, adolescents, and young adults with Down syndrome, as well as their associations with age, gender, and body weight. Methods: The research was conducted using the CAWI method among 310 caregivers of individuals with DS in Poland. Two validated tools were employed: the Montreal Children’s Hospital Feeding Scale (MCH-FS) and the Food Neophobia Scale for Children (FNSC). Body mass index (BMI), comorbidities, and demographic data were also analyzed. Results: Findings revealed that the majority of participants (55.2%) had normal body weight, while 19.4% were undernourished and 6.5% were classified as obese. Feeding difficulties of moderate to very high severity were reported in 26.5% of the participants. A high risk of food neophobia was identified in 41.3% of respondents, most frequently in the preschool age group. A statistically significant association was observed between age and the severity of both feeding difficulties and neophobia (*p* < 0.05). However, no significant relationships were found with gender or body weight. Conclusions: Feeding difficulties and food neophobia are prevalent among individuals with Down syndrome, particularly in preschool-aged children. The findings highlight the necessity of an interdisciplinary therapeutic approach and the individualization of dietary interventions, taking developmental age into account. Further studies are warranted, with consideration of environmental and psychosocial factors.

## 1. Introduction

Food neophobia, defined as reluctance to try new foods, may lead to nutritional deficiencies and complicate dietary management—especially in individuals with Down syndrome (DS), who often present with oral-motor dysfunction [1]. It is characterized by a child’s rejection of new or unfamiliar foods, both visually and in terms of taste [2,3,4].

The result of food neophobia can be a deficiency in some essential nutrients, especially vitamins and minerals. A number of studies confirm this, showing that the nutrition of children with high-level neophobia is tends to display insufficient variety. Children with food neophobia eat smaller amounts of vegetables, fruits, and dairy products than recommended. In addition, children who eat only selected foods may not develop the skills associated with fast eating, especially when they eat only soft or mushy foods [4,5,6,7,8,9].

Food neophobia makes it much more difficult for children with food allergies or intolerances, those who are overweight or obese, and those with medical conditions that require unique dietary approaches. These children are forced to adhere to an appropriate diet, and reluctance to consume new foods prevents the implementation of dietary recommendations, which can consequently affect the course of the disease. Although adequate nutrition is essential, it cannot fully reverse the effects of missed opportunities for optimal physical and cognitive development during early childhood [4,10,11]

In the diagnosis process related to feeding difficulties, using the Montreal Children’s Hospital Feeding Scale (MCH-FS) can be helpful. The Child Feeding Related MCH Scale is the first to be used for screening children with feeding difficulties, both for prevention and diagnosis. It is a perfect tool for determining possible feeding difficulties in children from 6 months to 6 years of age [12,13,14]. The Food Neophobia Scale for Children (FNSC), used to determine the level of food neophobia in young children, can also be helpful in practice. Both questionnaires were employed [5,15].

To date, very few studies have been published on food neophobia among children with DS, and they offer only a vague outline of the problem. Clavert et al. found that about half of the children with DS ate less than their energy requirements and consumed foods of low variety and small amounts of fruits and vegetables [16]. Hopman et al. showed that a Dutch group of children with DS had an increased incidence of consistency problems due to oral motor delays compared to children developing typically [17].

DS is a specific genetic defect characterized by the occurrence of various ailments, including precisely from the gastrointestinal tract. Proper nutrition for people with DS is essential for proper body development and helps prevent and treat many diseases. Efforts should be made to minimize as much as possible, and preferably remove, all difficulties associated with the nutrition of children and adults with DS [18].

Children with DS represent a population with specific and complex nutritional needs arising from the interplay of genetic predispositions, anatomical and physiological abnormalities, and a high prevalence of coexisting medical conditions. One of the most significant metabolic challenges is a reduced total energy expenditure, estimated to be on average 10–15% lower than in neurotypical children. This reduction, in combination with muscle hypotonia, low levels of physical activity, and decreased lean body mass, contributes to an increased risk of overweight and obesity [19,20]. Furthermore, the presence of endocrine disorders (e.g., hypothyroidism), autoimmune diseases (e.g., coeliac disease), and gastrointestinal disorders (e.g., gastroesophageal reflux, chronic constipation) necessitates not only dietary modifications but also regular monitoring of biochemical parameters and careful balancing of nutrients such as iron, selenium, iodine, dietary fiber, and B vitamins [21,22,23,24,25,26]. Many children with DS also exhibit immune dysfunction, which predisposes them to recurrent infections that may lead to temporary decreases in appetite and require adjustments in both the consistency and nutritional composition of meals [26].

Feeding difficulties—affecting up to 80% of children with DS—constitute another critical component of nutritional care [27]. Reduced oral muscle tone, macroglossia, impaired coordination of sucking, swallowing, and breathing, as well as delays in the development of fine motor skills and praxis, contribute to challenges in achieving feeding independence and in expanding dietary variety [28,29,30,31,32,33,34]. The high prevalence of food selectivity and neophobia—often associated with sensory hypersensitivity, fear of choking, and a strong preference for routine—can severely limit dietary diversity and increase the risk of micronutrient deficiencies [17,35,36]. Despite these complex needs, children with DS rarely receive specialized dietary care. A cross-sectional study conducted in Poland showed that 94.4% of children with DS were not under the care of a dietitian, despite nearly 20% having overweight or obesity and a markedly low intake of fruits and vegetables [18]. Moreover, the unnecessary use of elimination diets, such as the exclusion of lactose without medical justification, may lead to secondary intolerances, nutrient deficiencies, and disturbances in gut microbiota balance [18]. Given the absence of condition-specific nutritional guidelines—either in the European ESPGHAN and EFAD recommendations or in the guidelines issued by the American Academy of Pediatrics—it is imperative to develop tailored dietary protocols that address the individual health status, functional abilities, developmental characteristics, and environmental context of children with DS. Clinical dietitians should play a central role within interdisciplinary care teams, ensuring comprehensive, evidence-based, and individualized nutritional support for this vulnerable population [18,37,38].

The main objective of this study was to investigate the risk of food neophobia and feeding difficulties, mainly among children, adolescents, and young adults with DS. To evaluate feeding difficulties and food neophobia, two independent standardized tests were conducted to assess the risk of food neophobia and feeding difficulties in subjects with DS and whether there is a correlation between the risk of food neophobia and gender, age group, and body weight in subjects with Down syndrome. In addition, the impact of food neophobia risk level on the frequency of consumption of selected food groups among individuals with DS was assessed.

## 2. Materials and Methods

### 2.1. Study Group

The survey was conducted among parents or legal guardians of individuals with DS using the computer-assisted web interview (CAWI) method. Links to the survey were distributed to parents and legal guardians of individuals with Down syndrome. Data were collected nationwide between 5 December 2022 and 23 May 2023.

The sampling procedure was designed in accordance with ethical principles for research involving human participants, as outlined in the Declaration of Helsinki. A structured questionnaire was used, and retrospective data were collected. Additionally, approval for publication of the results was obtained from the Bioethics Committee of the Silesian Medical University in Katowice. A positive opinion was issued by the Committee for the study entitled “Feeding disorders and oral hypersensitivity in the context of sensory feeding in patients with neurodevelopmental disorders” (Resolution No. PCN/CBN/0052/KB1/142/22/23, approval date: 28 February 2023).

### 2.2. Inclusion Criteria

The inclusion criteria for the study comprised legal guardianship of a person with DS, provision of informed consent to participate by both the legal guardian and the child diagnosed with DS, and correct and complete completion of the questionnaire.

The exclusion criteria included enteral or parenteral feeding of the child, cases in which the parent or legal guardian was not responsible for the child’s nutrition (e.g., the child being in institutional care), as well as incorrect or incomplete completion of the questionnaire.

The survey form was intended solely for parents or legal guardians of individuals with DS, regardless of age. Following the application of the inclusion and exclusion criteria, a total of 310 parent/legal guardian–child pairs were included in the final data analysis.

### 2.3. Research Tool

As a research tool, a questionnaire was employed, incorporating two standardized instruments: the Montreal Children’s Hospital Feeding Scale (MCH-FS) and the Food Neophobia Scale for Children (FNSC), aimed at assessing food neophobia and feeding difficulties among children.

In the present study, the MCH-FS—originally developed by the Montreal Children’s Hospital Pediatric Feeding Program to evaluate feeding difficulties in children aged 6 months to 6 years—was utilized. Given the specific characteristics of the study population, which included children, adolescents, and young adults with Down syndrome, the tool was deemed appropriate for use across all age groups included in the study.

Individuals with DS frequently experience chronic feeding challenges, such as oral-motor dysfunction, food selectivity, oral hypersensitivity, and reduced appetite, which may persist throughout development. Consequently, although the MCH-FS was initially designed for younger children, it was applied in this study as a screening instrument for feeding difficulties across the entire cohort [5,12,13,14,15].

In the first part of the questionnaire, sociodemographic data of the parents or legal guardians were collected, including gender, age, maternal age at delivery, and level of education. The second part focused on anthropometric and perinatal data of the children, such as gender, gestational age at birth, chronological age (in years and months), height/length, and body weight.

Within this section, information was also gathered on comorbid conditions commonly observed in individuals with DS, including type 1 (insulin-dependent) diabetes mellitus, type 2 (non-insulin-dependent) diabetes mellitus, hypothyroidism, hyperthyroidism, Hashimoto’s disease, Graves–Basedow disease, celiac disease, esophageal atresia, duodenal atresia, anal atresia, Hirschsprung’s disease, lactose intolerance, oropharyngeal dysphagia (upper, transesophageal), and esophageal dysphagia (lower segment).

Additional questions addressed the presence of food allergies, cleft lip and/or palate, malocclusion, and the impact of dental abnormalities on feeding behavior.

Based on the data obtained [child’s weight, child’s height/length, and age], the body mass index (BMI) of the participants with DS was analyzed using established guidelines developed specifically for children with DS [39,40]. Appropriate percentile grids derived from scientific publications comparing BMI with age were applied to determine the BMI percentile for individuals aged 2 to 20 years [39]. For participants under 2 years of age, weight-for-age centile grids validated for children with DS were used [40].

Where guidelines were available, the referenced publications did not define rigid percentile thresholds for interpreting body weight. Therefore, the following BMI classifications were adopted for this study: a value below the 5th percentile was considered underweight; 5th–9th percentile as the lower limit of normal; 10th–75th percentile as normal; 75th–90th percentile as the upper limit of normal; 91st–95th percentile as overweight; and above the 95th percentile as obese. Although some sources define >80th percentile as indicative of being overweight, this approach was deemed methodologically inappropriate, as the available recommendations emphasize the 75th and 90th percentiles; thus, using the 80th percentile is regarded as only an approximate estimate and was not adopted in this analysis [40].

For participants aged over 20 years, standard adult BMI classification was applied, according to the World Health Organization (WHO) guidelines [41]: <18.5 kg/m^2^—underweight; 18.5–24.99 kg/m^2^—normal weight; 25.0–29.99 kg/m^2^—overweight; and ≥30.0 kg/m^2^—obesity.

The next part of the study focused on the assessment of dietary habits. Participants were asked about the frequency of consumption of selected food items and beverages using the standardized KomPAN questionnaire, which had been custom-modified to include additional food products not originally covered in the KomPAN tool [42].

Feeding difficulties in children were subsequently assessed using standardized research tools designed to evaluate food neophobia and problematic feeding behaviors. The instruments applied included the Children’s Food Neophobia Scale (FNSC) and the Montreal Children’s Hospital Feeding Scale (MCH-FS), developed by the Montreal Children’s Hospital Pediatric Feeding Program. In this study, the Polish version of the MCH-FS was used, having been appropriately translated and validated for the target population [5,12,13,14,15].

The Montreal Children’s Hospital Feeding Scale (MCH-FS), developed by the Pediatric Feeding Program, is designed to assess feeding behavior in children aged 6 months to 6 years who are receiving a mixed diet. However, due to the specific characteristics of the study population, the tool was applied across the entire cohort, including children, adolescents, and young adults with DS.

The scale consists of 14 questions addressing various aspects of feeding behavior. Examples of items include the following: “How do you rate your child’s meal pattern?”, “How concerned are you about your child’s meal pattern?”, “How do you rate your child’s appetite (feeling of hunger)?”, “At what point in the meal does your child start refusing to eat?”, “How long do your child’s meals last (in minutes)?”, “How do you rate your child’s behavior during meals?”, “Does your child whoop, choke, urinate, or vomit at certain foods?”, “Does your child hold food in his/her mouth without swallowing?”, “Do you have to walk behind your child or distract him/her (with toys, TV) to get him/her to eat?”, “Do you have to force your child to eat or drink?”, “How do you assess your child’s chewing (or sucking) skills?”, “How do you assess your child’s growth (weight, height)?”, “How does feeding your child affect your relationship with your child?”, “How does feeding your child affect your family relationship?” [12,13,14].

The scale evaluates specific domains, including oral motility [items 8 and 11], oral sensitivity [items 7 and 8], and appetite [items 3 and 4]. Additional questions explore caregivers’ concerns about feeding [items 1, 2, and 12], mealtime behavior [items 6 and 8], feeding strategies used by caregivers [items 5, 9, and 10], and the impact of feeding on family dynamics [items 13 and 14]. Each question is rated on a 7-point Likert scale.

The interpretation of the total MCH-FS score is as follows: 14–45 points—no feeding difficulties; 46–52 points—moderate difficulties; 53–58 points—moderate-to-severe difficulties; >59 points—severe difficulties [12,13,14].

For the Food Neophobia Scale (FNS) questionnaire, we used the 10 items of the original FNS developed by Pliner and Hobden [5,15], back-translated. Since the FNS referred to children, we used the Children Food Neophobia Scale (FNSC). The statements in the FNSC questionnaire were worded by adding the prefix “my child”. In the rest of this article, we use the abbreviation FNSC. We used the following statements: My child tries new and different foods all the time; My child doesn’t trust new foods; If my child doesn’t know what a particular food contains, he or she won’t try it; My child likes foods from different countries; My child finds ethnic foods (minorities and ethnic groups) too strange to eat; My child tries new foods; My child is afraid to eat things he or she has never eaten before; My child is very picky about the food we eat; My child will eat almost anything; My child would like to eat foods from other regions of Poland or other countries. In the FNSC, each item is rated on a 7-point agreement scale, from 1 = strongly disagree to 7 = strongly agree. All food neophilia statements were reversed so that the above scores indicate food neophobia. The total FNSC score was used to assess a person’s food neophobia level and propensity to try unfamiliar foods. Environmental, cultural, and social factors can influence eating behavior, so some statements were altered during translation of the scale to fit the appropriate cultural context and were adapted to the Polish language [43]. Points were converted according to a formula, and the score was interpreted according to the scale (≤27 pts.—low risk of food neophobia; 28–40 pts.—medium risk of food neophobia; ≥40 points—high risk of food neophobia).

For the purposes of this study, the 10 items from the original Food Neophobia Scale (FNS), developed by Pliner and Hobden [5,15], were used. The scale was back-translated to ensure linguistic and conceptual equivalence. Since the original FNS was designed for use in children, the adapted version—the Children’s Food Neophobia Scale (FNSC)—was applied. In this version, each phrase was rephrased by adding the prefix “my child”. Throughout this article, the abbreviation FNSC is used.

The following items were included in the FNSC: My child tries new and different foods all the time; My child doesn’t trust new foods; If my child doesn’t know what a particular food contains, he or she won’t try it; My child likes foods from different countries; My child finds ethnic foods (minorities and ethnic groups) too strange to eat; My child tries new foods; My child is afraid to eat things he or she has never eaten before; My child is very picky about the food we eat; My child will eat almost anything; My child would like to eat foods from other regions of Poland or other countries.

Each item was rated on a 7-point Likert scale (1 = strongly disagree; 7 = strongly agree). Statements expressing food neophilia were reverse-coded so that higher scores reflected a greater level of food neophobia. The total FNSC score was calculated to assess the overall degree of food neophobia and the child’s willingness to try unfamiliar foods.

Due to the influence of environmental, cultural, and social factors on eating behavior, selected items were adapted during the translation process to ensure cultural relevance and linguistic appropriateness for the Polish context [15,43]. Final scores were calculated using a standard formula, and interpreted as follows (Table 1): ≤27 points—low risk of food neophobia; 28–40 points—medium risk of food neophobia; ≥40 points—high risk of food neophobia [15,43].

### 2.4. Statistical Analysis

Microsoft Office Word, Microsoft Office Excel, and Statistica 13 were used for data analsis. The Chi-square test was applied to assess differences between qualitative variables, and *p*-values as well as the V-Cramer correlation coefficient were calculated. Friedman’s ANOVA test and Kendall’s coefficient of concordance were used to evaluate the relationships between gender, age, and FNSC scores. A statistical significance level of *p* ≤ 0.05 was adopted.

## 3. Results

### 3.1. Characteristics of the Studied Children with Down Syndrome

The study included 310 children and young adults with DS. Analysis of the data indicated that the group consisted of 169 males (54.52%) and 141 females (45.48%). The mean age of the participants was 7.55 years, with a minimum age of 4 months and a maximum age of 25 years and 10 months; the median age was 6.08 years.

Due to the broad age range, the participants were categorized into the following age groups: nursery-aged children (4 months to 2 years and 11 months), comprising 69 individuals (22.3%); preschool-aged children (3 years to 6 years and 11 months), comprising 101 individuals (32.6%); school-aged children (7 years to 10 years and 11 months), comprising 68 individuals (21.9%); adolescents (11 years to 17 years and 11 months), comprising 54 individuals (17.4%); and young adults (18 to 26 years), comprising 18 individuals (5.8%).

Body weight was assessed based on guidelines specifically developed for children and adolescents with DS.

The analysis of weight classification across different age groups of individuals with DS revealed that the most prevalent category was normal weight, observed in 55.16% of participants. Underweight was identified in 19.35% of the sample, the lower limit of normal weight in 11.94%, the upper limit of normal in 4.19%, overweight in 2.90%, and obesity in 6.45%. Underweight was most frequently observed in the preschool-aged group (25.74%), overweight in the young adult group (38.89%), and obesity also in the young adult group (27.78%). Statistical analysis was performed using Pearson’s Chi-square test. A *p*-value of 0.00 indicated statistically significant differences in body weight distribution across age groups. The V-Cramer correlation coefficient of 0.31 suggested a moderate association between age group and body weight classification (Table 2).

### 3.2. Complaints and Diseases Associated with Down Syndrome in the Study Group

Comorbidities and symptoms experienced daily by children, adolescents, and young adults with DS in the study group were analyzed. A total of 99 respondents (31.9%) reported that their child had no additional health conditions, while 150 respondents (48.4%) indicated the presence of one disease or condition, and 61 respondents (19.7%) reported more than one comorbidity.

The most frequently reported condition was hypothyroidism, diagnosed in 166 individuals (53.5%). Lactose intolerance was the most common intolerance, affecting 45 individuals (14.5%). Hashimoto’s disease was reported in 20 individuals (6.5%), hyperthyroidism in 10 (3.2%), celiac disease in 7 (2.3%), and duodenal atresia in 8 (2.6%). Esophageal dysphagia (lower) was present in six individuals (1.9%), while oropharyngeal dysphagia (upper, transesophageal) and Hirschsprung’s disease were each diagnosed in five individuals (1.6%). Type 1 (insulin-dependent) diabetes mellitus affected two individuals (0.6%), and type 2 (non-insulin-dependent) diabetes mellitus, anal atresia, and Graves–Basedow disease were each observed in one individual (0.3%). No cases of esophageal atresia were reported.

Regarding craniofacial abnormalities, three individuals (1.0%) had an active cleft lip at the time of the study, and two individuals (0.6%) had undergone surgical correction. The remaining respondents reported no cleft lip. A cleft palate was observed in 10 individuals (3.2%) at the time of the study, and 3 individuals (1.0%) had a corrected cleft palate. Additionally, two individuals (0.6%) had a history of both cleft lip and cleft palate that had been surgically corrected prior to the study.

In terms of malocclusion, 195 participants (62.9%) had no malocclusion at the time of assessment. Malocclusion was present in 110 individuals (35.5%), and 5 individuals (1.6%) had a corrected malocclusion. Among those with active malocclusion (n = 110), 12 respondents (10.9%) reported that the condition significantly interfered with eating, whereas 98 (89.1%) indicated that eating was not affected.

Food allergies were absent in 263 children (84.8%). The most frequently reported allergy was to cow’s milk protein (19 individuals, 6.1%). Other less common allergies included apple, pear, carrot, celery, peach, plum, tomato, cherry, melon, watermelon, romaine, wheat, cauliflower, zucchini, cocoa, dill, honey, nuts, blackberry, baker’s yeast, rice, apricot, tangerine, orange, lemon, citrus fruits, banana, strawberries, raspberries, soy, gluten, pineapple, olives, kiwi, bee pollen, corn, chicken egg, egg yolk, fish, yams, parsley, peppers, carotene, and goat’s milk protein.

### 3.3. Feeding Difficulties in the Study Group According to the MCH-FS Scale

The level of feeding difficulty was assessed using the Polish version of the Montreal Children’s Hospital Feeding Scale (MCH-FS). The results were analyzed according to age group and gender.

The mean MCH-FS score among individuals with DS was 36.6 points for females (median = 34) and 36.5 points for males (median = 34). The highest mean scores were observed in the preschool-aged group, with 40.2 points in females (median = 40) and 40.8 points in males (median = 41). The lowest mean scores were recorded in the young adult group, with 25.1 points for females (median = 23) and 27.8 points for males (median = 28).

Statistical analysis was conducted using Friedman’s ANOVA test, Kendall’s coefficient of concordance, and the Chi-square test. The Friedman’s ANOVA test yielded the following results: ANOVA (N = 310, *df* = 2) = 244.6624, *p* = 0.0000. The concordance coefficient was 0.39426, and the mean rank correlation was 0.039266.

Based on the results, a statistically significant difference was found between MCH-FS scores and participants’ age group and gender, as determined by Friedman’s ANOVA test. Furthermore, moderate agreement between the variables was indicated by Kendall’s coefficient of concordance (Table 3).

Difficulties in feeding were assessed using the MCH-FS score and analyzed in relation to age group and gender. The results of the analysis indicated a statistically significant association between the MCH-FS score and both the age group and gender of individuals with DS (Table 4).

### 3.4. Testing the Risk of Food Neophobia

The Food Neophobia Scale for Children (FNSC) was used to assess the risk of food neophobia in the study group. The mean score in the total sample of individuals with Down syndrome was 37.1 ± 16.4, with a median of 36, a minimum of 10, and a maximum of 70 points.

The highest average scores were observed in the preschool-aged group, with 42.6 points in females and 44.8 points in males. The lowest mean score was recorded among females in the nursery-aged group, amounting to 20.8 points (Table 5).

Statistical analysis of the above data was conducted using Friedman’s ANOVA test, with the calculation of Kendall’s coefficient of concordance and the Chi-square test. The results were as follows: ANOVA (n = 310, *df* = 2) = 122.9066, *p* = 0.0000; concordance coefficient = 0.19824; mean rank correlation = 0.19564.

The result of Friedman’s ANOVA test (*p* = 0.0000) indicated that both gender and age have a statistically significant effect on FNSC scores. A significant association was observed between these variables. Kendall’s coefficient of concordance (0.19824) suggested a moderate level of agreement between gender, age, and FNSC scores. Although a relationship was detected, its strength was limited.

When the risk of food neophobia was compared by gender, the *p*-value of 0.000 indicated a statistically significant difference in food neophobia risk in relation to both age and gender.

Table 6 presents the distribution of food neophobia risk in the study group of individuals with DS according to gender. The analysis revealed that a low risk of food neophobia was observed in 34.19% of participants, with a higher prevalence among females (38.30%) compared to males (30.77%). A moderate risk was identified in 24.52% of the participants, with a comparable distribution between genders (24.82% in females and 24.26% in males). A high risk of food neophobia was found in 41.29% of the participants, with a higher prevalence among males (44.97%) than females (36.88%). No statistically significant differences were found between gender groups (*p* = 0.29).

Table 7 presents the distribution of food neophobia risk among individuals with DS across different age groups. The analysis revealed statistically significant differences in the level of risk according to age (*p* < 0.01). A low risk of food neophobia was most frequently observed among young adults (72.22%) and nursery-aged children (47.83%), whereas the lowest percentage of individuals at low risk was found among preschool-aged children (19.80%). The highest proportion of individuals at high risk of food neophobia was identified in the preschool-aged group (57.43%). The correlation coefficient was 0.26, indicating a weak but statistically significant association between age and the risk of food neophobia.

In addition, the relationship between body weight and the presence of food neophobia was assessed in the study group. According to both the MCH-FS and FNSC scales, *p*-values exceeded 0.05, indicating no statistically significant correlation between body weight classification and food neophobia risk among individuals with Down syndrome.

A high risk of feeding difficulties was more frequently observed among individuals with obesity (15%) and those at the upper limit of normal weight (30.8%); however, this association was not statistically significant (*p* = 0.29) (Table 8).

Similarly, a high risk of food neophobia was more prevalent in individuals with obesity (55%) and those at the upper limit of normal weight (53.8%), and lowest among those classified as overweight (22.2%). This association also did not reach statistical significance (*p* = 0.22) (Table 9).

Table 10 presents the results concerning the frequency of consumption of selected food groups, taking into account the level of food neophobia risk. Individuals identified as being at high risk of food neophobia were significantly more likely to avoid foods with high nutritional value and complex sensory characteristics.

In this group, the lowest consumption frequencies were observed for the following products: vegetables (*p* = 0.00; *r* = 0.24), fish (*p* = 0.00; *r* = 0.22), legumes (*p* = 0.00; *r* = 0.21), nuts (*p* = 0.00; *r* = 0.21), flavored cottage cheese (*p* = 0.01; *r* = 0.19), wholemeal bread (*p* = 0.03; *r* = 0.18), and red meat dishes (*p* = 0.03; *r* = 0.18).

Only 18.0% of individuals in the high-risk group reported consuming vegetables several times per day, compared to 48.1% in the low-risk group. In the case of fish, 16.4% of those at high risk reported never consuming them, whereas in the low-risk group, this applied to only 2.8%. Legume consumption was particularly low among high-risk individuals, with 76.6% consuming them less than once a week, including 37.5% who did not consume them at all.

Nuts were avoided by 62.5% of high-risk participants, in contrast to 34.0% in the low-risk group. Interestingly, a slightly higher proportion of individuals in the high-risk group consumed flavored cottage cheese once or several times a week (16.4% and 14.1%, respectively) compared to the low-risk group (11.3% and 10.4%, respectively).

Regarding wholemeal bread, 50.0% of high-risk individuals reported no consumption, compared to 35.8% in the low-risk group. Moreover, 23.6% of the low-risk group consumed wholemeal bread several times a week. Similarly, red meat dishes were avoided more often in the high-risk group (28.9% reporting no consumption) than in the low-risk group (10.4%).

For other food categories—such as white bread, milk, fermented dairy beverages (both natural and flavored), white meat, processed meats, sweets, instant soups, and canned foods—no statistically significant differences were observed between the groups (*p* > 0.05). However, some trends were noted. For example, fruit consumption approached statistical significance (*p* = 0.0547; *r* = 0.17), suggesting a potential tendency for reduced intake among individuals with higher neophobia scores.

## 4. Discussion

Nutrition in individuals with DS represents a highly complex and multifactorial issue, shaped by the presence of numerous comorbidities commonly associated with trisomy 21. Adequate dietary management is a critical component of care in this population, as it plays a pivotal role in both the prevention and clinical management of conditions to which individuals with DS are more susceptible compared to the general population. Moreover, appropriate nutritional strategies may contribute to mitigating the impact of existing comorbidities.

Despite its clinical relevance, there is a marked paucity of scientific literature specifically addressing the nutritional needs of individuals with DS. This gap may be partly explained by the relatively low prevalence of the condition, which poses challenges to the design and execution of large-scale, high-quality studies. Consequently, the development of evidence-based dietary recommendations tailored to this population remains limited. Additionally, the nutritional care of individuals with DS is inherently complex and methodologically demanding, further contributing to its underrepresentation in current research.

The present study employed the CAWI method, a widely recognized and effective approach for collecting large datasets, particularly from populations that are typically difficult to access. This methodology enabled the recruitment of a substantial sample of 310 individuals with DS, thereby enhancing the reliability of the statistical analyses and offering valuable insights into a population that remains underrepresented in the scientific literature.

As previously noted, the number of contemporary publications addressing the nutritional status and feeding behaviors of individuals with DS remains limited. Consequently, some of the sources referenced in this study include older publications whose findings are nonetheless still considered relevant.

Available literature consistently indicates that individuals with DS are at increased risk of overweight and obesity [19]. However, this trend was not observed in the current study. The majority of participants (n = 171; 55.2%) exhibited a normal body weight, while 60 individuals (19.4%) were classified as underweight. Only nine participants (2.9%) met the criteria for overweight, and 6.5% were classified as obese. Previous research has reported a particularly high prevalence of excessive weight gain among children with DS aged 4–5 years [44], a finding that was not corroborated by the present dataset.

A statistically significant association was identified between body weight classification and age group, as confirmed by the conducted statistical analyses. These findings indicate that the distribution of body weight among individuals with DS varies significantly across different age groups.

In the context of common metabolic and endocrine comorbidities—such as hypothyroidism, celiac disease, and various food intolerances—frequently observed in individuals with DS, limited acceptance of novel foods may represent a considerable barrier to the effective implementation of dietary interventions and nutrition-related medical recommendations.

Although statistical analyses did not reveal a significant association between body weight and the severity of either feeding difficulties or food neophobia, it is notable that some individuals classified as overweight or obese presented with elevated levels of feeding difficulty. This observation may reflect the use of compensatory feeding strategies by caregivers, involving the repeated provision of a narrow selection of high-calorie, preferred foods. Such practices may contribute to excessive energy intake while simultaneously reducing dietary diversity.

Food neophobia refers to the tendency to avoid or reject unfamiliar or novel foods. Its manifestation, particularly during early childhood, can substantially affect food selection, the development of taste preferences, and overall dietary quality. From an evolutionary standpoint, food neophobia may serve a protective role by reducing the likelihood of ingesting potentially harmful substances. However, this same protective mechanism may also impede the exploration and acceptance of nutritionally valuable foods.

In contemporary contexts characterized by food safety and controlled environments, the adaptive function of food neophobia may be diminished, although a cautious approach to novel foods remains common. A strong correlation has been documented between food neophobia and reduced dietary diversity, as well as limited prior exposure to a variety of foods [4].

Despite its relevance to dietary intake and nutritional health, food neophobia remains a relatively underexplored area in the scientific literature. One earlier study reported that approximately 50% of children with DS consumed less energy than recommended and demonstrated a limited variety of food intake, particularly with respect to fruits and vegetables [16]. Another study found that children with DS experienced more frequent difficulties related to food texture, which were attributed to delays in oral-motor development compared to typically developing peers [17].

In the present study, two standardized assessment tools were utilized to evaluate feeding difficulties and the risk of food neophobia. Both instruments were administered to all participants, irrespective of age. Given the cognitive limitations commonly associated with trisomy 21, the questionnaires were completed by the parents or legal guardians of the individuals with DS. Accordingly, the use of statements in the first-person possessive form (e.g., “my child…”) was deemed appropriate and consistent with the methodology.

Results obtained using MCH-FS indicated that the majority of individuals with DS exhibited minimal or no feeding difficulties. Moderate difficulties were reported in 42 participants (13.5%), high levels of difficulty in 19 participants (6.1%), and very high levels in 21 participants (6.8%). In total, 82 individuals (26.5%) were classified as having moderate to very high levels of feeding difficulties.

According to FNSC, 76 individuals (24.5%) were categorized as being at moderate risk of food neophobia, while 128 individuals (41.3%) were identified as being at high risk.

The interpretation of both assessment tools clearly suggests that food neophobia is a prevalent issue among individuals with DS. Statistical analyses further revealed a significant association between both feeding difficulties and food neophobia with age group and gender.

In population-based research conducted in Poland, approximately 10% of children have been reported to exhibit high levels of food neophobia, with the highest prevalence observed in children aged 5–6 years. Most studies to date indicate that food neophobia is not significantly associated with gender [4,15].

The findings of the present study offer valuable insights into the prevalence and determinants of feeding difficulties and food neophobia among children and young adults with DS. Particularly noteworthy are the observed variations in the severity of these challenges across different age groups and genders, which align with existing literature indicating the highest levels of food neophobia in preschool-aged children [4,5].

Food neophobia, defined as an aversion to trying unfamiliar foods, can significantly impact dietary diversity and overall nutritional quality. In the present study population, a substantial proportion of individuals with DS were classified as being at medium to high risk of food neophobia. This condition may have long-term nutritional implications, particularly in relation to insufficient intake of essential micronutrients, including vitamins, minerals, and dietary fiber [4,5,6,7,8,9].

These findings are consistent with previous studies reporting that children with selective or restrictive food repertoires often consume energy-dense but nutrient-poor diets. Such dietary patterns may contribute to the development of overweight or obesity, despite the presence of feeding difficulties [6,8].

The results of the present study also revealed that feeding difficulties in individuals with DS are not limited to food refusal. They may also encompass atypical peri-meal behaviors, such as prolonged mealtimes, the necessity of distraction during feeding, and defensive reactions including whooping, gagging, choking, or vomiting reflexes. These disturbances may negatively impact family dynamics and contribute to the emergence of secondary feeding disorders and developmental delays.

Given the multifactorial nature of these challenges, dietary management in individuals with DS should adopt an interdisciplinary approach. Effective intervention requires collaboration among professionals such as speech and language therapists, sensory integration specialists, child psychologists, and clinical dietitians.

An important observation emerging from this study is the lack of strong correlations between the presence of comorbidities and the severity of food neophobia or feeding difficulties. This finding may be explained by the nature of certain medical conditions—such as celiac disease or lactose intolerance—which necessitate dietary restrictions and may, therefore, indirectly influence eating behaviors and attitudes toward food. At the same time, caregivers may successfully compensate for these conditions, thereby reducing their apparent impact on the outcomes of standardized screening tools.

From the perspective of clinical nutrition and dietetic practice, it is essential to account for comorbidities when planning individualized nutritional interventions and assessing the risk of nutrient deficiencies.

Despite the use of screening tools originally developed for younger children, their application across the entire study population—including adolescents and young adults—should be viewed as a valuable aspect of this research. This approach is justified by the underrepresentation of older individuals with DS in studies addressing nutritional concerns, despite the persistence of maladaptive eating behaviors and limited food acceptance beyond early childhood. Accordingly, the findings of the present study may serve as a foundation for the development of more sophisticated diagnostic tools tailored to the developmental characteristics and nutritional needs of this population.

An additional area warranting further investigation is the influence of environmental and familial factors, including caregiver education, feeding styles, dietary beliefs, food availability, and prior experience with dietary interventions. Incorporating these variables into future research could provide a more comprehensive understanding of the mechanisms underlying dietary behavior in individuals with DS. Moreover, such an approach would support the development of more personalized and effective dietary therapeutic strategies.

The interpretation of the above results should be undertaken with consideration of the study’s strengths and limitations. The present study offers a valuable contribution to the growing body of knowledge concerning feeding difficulties and food neophobia in children and young adults with DS—an area that remains relatively underexplored in the scientific literature. The large sample size (n = 310) and broad age range allowed for statistically robust analyses and facilitated the identification of significant associations between age, comorbidities, and the severity of feeding challenges.

A particular strength of the study was the use of standardized and culturally adapted assessment instruments—MCH-FS and FNSC—which enhanced the validity and reliability of the data collected. Additionally, the application of syndrome-specific growth reference charts for children and adolescents with DS enabled a more accurate assessment of nutritional status and minimized the risk of misclassification.

The study was conducted in accordance with ethical research principles, having received a favorable opinion from the Bioethics Committee of the Silesian Medical University. The use of the CAWI technique facilitated participant recruitment from diverse geographic regions, thereby increasing the representativeness of the sample. Statistical analyses were performed using appropriate tests and coefficients selected according to the type and distribution of the variables, which further strengthened the credibility and interpretability of the findings.

Despite its numerous strengths, the present study has certain limitations that should be considered when interpreting the results. Firstly, the psychometric tools employed—although validated—were originally developed for preschool- and early school-aged children. Their application to adolescents and young adults may reduce measurement precision within this subgroup. Nevertheless, the inclusion of older participants represents a significant step toward addressing a notable gap in the literature, as this population is often underrepresented in studies on feeding difficulties and eating behaviors.

Secondly, the use of caregiver-reported questionnaires introduces the potential for subjective bias and cognitive distortion, which may limit the objectivity of the collected data. Additionally, the absence of a control group (e.g., neurotypical peers) precludes conclusions regarding the specificity of the observed difficulties to individuals with DS. Although data on comorbidities, food allergies, and malocclusion were collected, their potential impact on the analyzed phenomena was not explored in depth, thereby limiting the ability to draw causal inferences.

Another limitation pertains to the lack of comparison between the study sample and national demographic data on individuals with DS, which may affect the generalizability of the findings. Furthermore, young adults constituted only 5.8% of the total sample, restricting the extent to which conclusions can be extrapolated to this age group.

Despite these limitations, the present study constitutes a valuable contribution to both theoretical and practical knowledge. The findings provide a foundation for future comparative and interventional research, and may inform the development of targeted strategies to support nutritional care and dietary therapy in individuals with DS. Future research should consider incorporating environmental and psychosocial variables—such as caregiver education, feeding style, and family mealtime dynamics—which are likely to play a pivotal role in shaping eating behaviors and caregiving strategies. This study offers a robust basis for further exploration within the fields of clinical nutrition, pediatric dietetics, and developmental neuropsychology.

## 5. Conclusions

The risk of food neophobia and feeding difficulties among individuals with DS varies significantly by age. The highest severity of feeding challenges—manifested both as avoidance of novel foods and atypical feeding behaviors—was observed in preschool-aged children. These difficulties tended to decrease with age, reaching the lowest levels among young adults.

Gender did not exert a statistically significant effect on the level of food neophobia, although males demonstrated slightly higher scores on the FNSC. These differences, however, did not reach statistical significance, suggesting comparable levels of neophobia between females and males with DS.

No significant associations were identified between body weight status and the severity of feeding difficulties or food neophobia. This indicates that individuals with DS—regardless of whether they were underweight, overweight, or obese—exhibited similar patterns of feeding challenges.

A statistically significant relationship was observed between age and the severity of feeding difficulties. The moderate strength of this association highlights the relevance of developmental stage in shaping feeding behaviors among individuals with DS. Accordingly, nutritional and therapeutic interventions should be tailored to the child’s or adolescent’s developmental profile.

The high prevalence of food neophobia observed in this population was associated with limited dietary diversity, particularly with respect to nutrient-dense food groups such as vegetables, fruits, fish, legumes, nuts, wholemeal bread, and red meat dishes. Individuals at higher risk of food neophobia were more likely to avoid these foods or consume them only occasionally. In contrast, those with low neophobia scores exhibited more balanced dietary patterns, characterized by frequent consumption of fruits and vegetables, higher intake of fermented and whole-grain products, and relatively lower consumption of ultra-processed foods.

The relationship between neophobia level and food choices has important clinical and practical implications, particularly for the design of dietary interventions, nutrition education, and behavioral therapy targeted at children and young adults with DS.

The high prevalence of comorbidities—such as hypothyroidism, food intolerances, and malocclusion—may further influence feeding behaviors in this population. Although the present study did not analyze their direct impact on food neophobia or feeding difficulties, these factors may play a modifying role and warrant further investigation.

## 6. Clinical Implications

1. Implementation of early nutritional intervention programs in kindergartens and at home: Given the high prevalence of food neophobia and feeding difficulties among preschool-aged children, it is recommended to develop and implement educational and intervention programs in both home settings and early education institutions. These programs should promote gradual exposure to new foods, the development of positive sensory experiences, and the cultivation of openness toward dietary diversity.
2. Regular assessment of eating patterns using structured screening tools: Due to the widespread occurrence of food selectivity and limited dietary variety, it is advisable to systematically employ standardized screening tools for the assessment of feeding difficulties and food neophobia in clinical practice—particularly in dietetic, pediatric, and rehabilitation settings that serve children with DS.
3. Incorporating psychosocial factors into dietary intervention planning: When designing nutritional interventions, it is essential to consider not only the child’s sensory preferences but also the caregiver’s feeding style, level of nutritional knowledge, and attitudes toward food. Interventions should be preceded by a comprehensive assessment of the home environment, including family mealtime dynamics and behavioral modeling practices.
4. Personalization of dietary recommendations in the context of coexisting medical and behavioral constraints: In cases where comorbid conditions require dietary restrictions (e.g., coeliac disease, food intolerances), it is recommended to develop personalized nutrition plans that reconcile medical requirements with the child’s individual sensory preferences regarding food texture, taste, and smell.
5. Applying interactive nutrition education methods tailored to cognitive developmental levels: When working with children with DS, interactive and action-based approaches—such as sensory food play, therapeutic cooking sessions, or acceptance training through play—may prove effective. The development of dedicated educational materials and structured lesson plans is recommended to support the formation of positive eating behaviors.
6. Monitoring dietary diversity as an indicator of intervention effectiveness: To comprehensively assess the outcomes of nutritional therapy, it is recommended to monitor not only anthropometric parameters such as body weight but also dietary diversity scores, with particular attention to the intake of vegetables, fruits, whole grains, and both plant- and animal-based protein sources.
7. Training interdisciplinary therapeutic teams on feeding difficulties in children with intellectual disabilities: The findings of this study highlight the need to incorporate topics related to feeding difficulties and food neophobia into professional training programs for healthcare providers working with children with DS—including pediatricians, speech and language therapists, sensory integration specialists, and clinical dietitians. A coordinated, interdisciplinary approach based on shared terminology and therapeutic goals is essential for delivering effective support to this population.

## Figures and Tables

**Table 1:** Scores on the Montreal Children’s Hospital Feeding Scale (MCH-FS) and the Food Neophobia Scale for Children (FNSC).

The Montreal Children’s Hospital Feeding Scale (MCH-FS)		The Children’s Food Neophobia Scale (FNSC)	
14–45 points	no feeding difficulties		
46–52 points	moderate difficulties	≤27 points	low risk of food neophobia
53–58 points	moderate-to-severe difficulties	28–40 points	medium risk of food neophobia
>59 points	severe difficulties	≥40 points	high risk of food neophobia

**Table 2:** Body weight of subjects with DS by age.

Weight	Age Group												*p*-Value	Correlation Coefficient
	Nursery Age n = 69		Preschool Age n = 101		School Age n = 68		Youth n = 54		Young n = 18		Total N = 310			
	n	%	n	%	n	%	n	%	n	%	N	%		
Underweight	12	17.39%	26	25.74%	14	20.59%	8	14.81%	0	0.00%	60	19.35%	*p* = 0.00	0.31
Lower limit of the standard	6	8.70%	13	12.87%	10	14.71%	8	14.81%	0	0.00%	37	11.94%		
Normal body weight	43	62.32%	53	52.48%	35	51.47%	34	62.96%	6	33.33%	171	55.16%		
Upper limit of the standard	3	4.35%	3	2.97%	4	5.88%	3	5.56%	0	0.00%	13	4.19%		
Overweight	0	0.00%	0	0.00%	1	1.47%	1	1.85%	7	38.89%	9	2.90%		
Obesity	5	7.25%	6	5.94%	4	5.88%	0	0.00%	5	27.78%	20	6.45%		

**Table 3:** Mean scores of the MCH-FS test assessing the risk of food neophobia by gender and age group (*p* = 0.0000).

Age Group	MCH-FS Test Result					
	$\bar{x}$ ± SD		Me		Min-Max.	
	Female	Male	Female	Male	Female	Male
Nursery age	38.4 ± 13.4	38.7 ± 14.1	35	35	17–70	15–70
Preschool age	40.2 ± 12.8	40.8 ± 12.6	40	41	15–65	15–67
School age	34.7 ± 11.9	37.2 ± 12.1	33	35	16–67	18–65
Youth	30.9 ± 10.6	30.3 ± 10.1	29	28	15–57	16–60
Adults	25.1 ± 8.9	27.8 ± 9.2	23	28	16–50	16–50
Total	36.6 ± 13.0	36.5 ± 12.9	34	34	15–70	15–70

$\bar{x}$ ± SD—arithmetic mean, standard deviation; ME—median; Min—minimum; Max—maximum.

**Table 4:** Level of feeding difficulties according to the MCH-FS scale of subjects with Down syndrome studied by age.

Level of Difficulty in Feeding	Age Group										Total N = 310		*p*-Value	Correlation Coefficient
	Nursery Age n = 69		Preschool Age n = 101		School Age n = 68		Youth n = 54		Young n = 18					
	n	%	n	%	n	%	n	%	n	%	N	%		
No difficulties or minor difficulties in feeding N = 228	53	76.81	62	61.39	53	76.81	48	88.89	17	94.44	228	73.55	0.00	0.19
Moderate difficulties in feeding N = 42	4	5.80	23	22.77	11	15.94	3	5.56	1	5.56	42	13.55		
Serious difficulties in feeding N = 19	7	10.14	9	8.91	1	1.45	2	3.70	0	0.00	19	6.13		
Very serious difficulties in feeding N = 21	10	14.49	7	6.93	3	4.35	1	1.85	0	0.00	21	6.77		

**Table 5:** Mean scores of the FNSC test assessing the risk of food neophobia by gender and age group (*p* = 0.000).

	FNSC Test Result					
Age Group	$\bar{x}$ ± SD		Me		Min-Max.	
	Female	Male	Female	Male	Female	Male
Nursery age	20.8 ± 12.2	29.6 ± 12.2	28	28	10–62	10–62
Pre-school age	42.6 ± 15.0	44.8 ± 14.8	42	45	10–70	16–70
School age	40.3 ± 18.1	41.9 ± 17.7	41	43	10–70	10–70
Youth	34.0 ± 15.3	34.5 ± 16.3	34	33	10–70	10–67
Adults	23.2 ± 11.5	21.9 ± 9.3	21	21	10–49	10–39
Total	37.3 ± 16.4	37.1 ± 16.4	36	36	10–70	10–70

$\bar{x}$ ± SD—arithmetic mean, standard deviation.

**Table 6:** Risk of food neophobia in subjects with Down syndrome by gender.

Risk of Food Neophobia	Gender of the Child				Total		*p*-Value	Correlation Coefficient
	Female		Male					
	n = 141	%	n = 169	%	N = 310	%		
Low risk of food neophobia n = 106	54	38.30	52	30.77	106	34.19	0.29	0.90
Average risk of food neophobia n = 76	35	24.82	41	24.26	76	24.52		
High risk of food neophobia n = 128	52	36.88	76	44.97	128	41.29		

**Table 7:** Risk of food neophobia in study subjects with Down syndrome by age group.

Risk of Food Neophobia	Age Group										*p*-Value	Correlation Coefficient
	Nursery Age n = 69		Preschool Age n = 101		School Age n = 68		Youth n = 54		Young n = 18			
	n	%	n	%	n	%	n	%	n	%		
Low risk of food neophobia N = 106	33	47.83	20	19.80	19	27.54	21	38.89	13	72.22	0.00	0.26
Average risk of food neophobia N = 76	22	31.88	23	22.77	13	18.84	14	25.93	4	22.22		
High risk of food neophobia N = 128	14	20.29	58	57.43	3	4.35	19	35.19	1	5.56		

**Table 8:** Comparison of risk of food neophobia versus body weight among subjects with Down syndrome according to MCH-FS test.

Weight Classification	Feeding Difficulties According to the MCH-FS Test				*p*-Value	Correlation Coefficient
	None or Low	Medium Risk	High Risk	Very High Risk		
Underweight n = 60	40 (66.7%)	7 (11.7%)	6 (10.0%)	7 (11.7%)	0.29	0.14
Lower limit of the standard n = 37	29 (78.4%)	4 (10.8%)	2 (5.4%)	2 (5.4%)		
Normal body weight n = 171	132 (77.2%)	23 (13.5%)	7 (4.1%)	9 (5.3%)		
Upper limit of the standard n = 13	8 (61.5%)	1 (7.7%)	2 (15.4%)	2 (15.4%)		
Overweight n = 9	8 (88.9%)	1 (11.1%)	0 (0.0%)	0 (0.0%)		
Obesity n = 20	11 (55.0%)	6 (30.0%)	2 (10.0%)	1 (5.0%)		

**Table 9:** Comparison of risk of food neophobia vs. body weight among subjects with Down syndrome according to FNSC test.

Weight Classification	Risk of Food Neophobia According to FNSC Test			*p*-Value	Correlation Coefficient
	Low Risk	Medium Risk	High Risk		
Underweight n = 60	20 (33.3%)	14 (23.3%)	26 (43.3%)	0.22	0.14
Lower limit of the standard n = 37	10 (27.0%)	11 (29.7%)	16 (43.2%)		
Normal body weight n = 171	61 (35.7%)	42 (24.6%)	68 (39.8%)		
Upper limit of the standard n = 13	4 (30.8%)	2 (15.4%)	7 (53.8%)		
Overweight n = 9	7 (77.8%)	2 (22.2%)	0 (0.0%)		
Obesity n = 20	4 (20.0%)	5 (25.0%)	11 (55.0%)		

**Table 10:** Frequency of consumption of selected food products by subjects with Down syndrome by risk of food neophobia according to FNSC questionnaire.

Food Products	Risk of Food Neophobia	Frequency of Consumption of Food Products						*p*-Value
		Never	1–3 Times a Month	Once a Week	Several Times a Week	Once a Day	Several Times a Day	
Light bread, such as wheat, rye, mixed wheat-rye, toast, rolls, croissants	Low	23	19	7	20	21	16	0.08
	N = 106	(21.7%)	(17.9%)	(6.6%)	(18.9%)	(19.8%)	(15.1%)	
	Averages	27	5	8	5	13	18	
	N = 76	(35.5%)	(6.6%)	(10.5%)	(6.6%)	(17.1%)	(23.7%)	
	High	32	14	8	19	26	29	
	N = 128	(25.0%)	(10.9%)	(6.3%)	(14.8%)	(20.3%)	(22.7%)	
Wholemeal bread	Low	38	13	11	25	13	6	0.03
	N = 106	(35.8%)	(12.3%)	(10.4%)	(23.6%)	(12.3%)	(5.7%)	
	Averages	38	14	8	5	4	7	
	N = 76	(50.0%)	(18.4%)	(10.5%)	(6.6%)	(5.3%)	(9.2%)	
	High	64	19	11	15	7	12	
	N = 128	(50.0%)	(14.8%)	(8.6%)	(11.7%)	(5.5%)	(9.4%)	
White rice, plain pasta or small groats, e.g., semolina, couscous	Low	9	21	33	40	3	0	0.09
	N = 106	(8.5%)	(19.8%)	(31.1%)	(37.7%)	(2.8%)	(0.0%)	
	Averages	9	14	19	26	8	0	
	N = 76	(11.8%)	(18.4%)	(25.0%)	(34.2%)	(10.5%)	(0.0%)	
	High	12	23	37	39	10	7	
	N = 128	(9.4%)	(18.0%)	(28.9%)	(30.5%)	(7.8%)	(5.5%)	
Buckwheat groats, oatmeal, whole-grain pasta or other coarse-grain groats	Low	15	17	22	41	9	2	0.20
	N = 106	(14.2%)	(16.0%)	(20.8%)	(38.7%)	(8.5%)	(1.9%)	
	Averages	14	14	17	22	8	1	
	N = 76	(18.4%)	(18.4%)	(22.4%)	(28.9%)	(10.5%)	(1.3%)	
	High	29	34	26	25	12	2	
	N = 128	(22.7%)	(26.6%)	(20.3%)	(19.5%)	(9.4%)	(1.6%)	
Fast food, such as French fries, hamburgers, pizza, hot dogs, casseroles	Low	41	56	7	1	1	0	0.22
	N = 106	(38.7%)	(52.8%)	(6.6%)	(0.9%)	(0.9%)	(0.0%)	
	Averages	40	34	2	0	0	0	
	N = 76	(52.6%)	(44.7%)	(2.6%)	(0.0%)	(0.0%)	(0.0%)	
	High	51	59	12	3	3	0	
	N = 128	(39.8%)	(46.1%)	(9.4%)	(2.3%)	(2.3%)	(0.0%)	
Fried foods (e.g., meat or flour)	Low	17	29	26	30	3	1	0.09
	N = 106	(16.0%)	(27.4%)	(24.5%)	(28.3%)	(2.8%)	(0.9%)	
	Averages	17	19	29	9	2	0	
	N = 76	(22.4%)	(25.0%)	(38.2%)	(11.8%)	(2.6%)	(0.0%)	
	High	17	33	33	42	3	0	
	N = 128	(13.3%)	(25.8%)	(25.8%)	(32.8%)	(2.3%)	(0.0%)	
Butter as an additive to bread or dishes. for frying, baking, for example.	Low	15	13	10	21	26	21	0.06
	N = 106	(14.2%)	(12.3%)	(9.4%)	(19.8%)	(24.5%)	(19.8%)	
	Averages	19	11	2	14	8	22	
	N = 76	(25.0%)	(14.5%)	(2.6%)	(18.4%)	(10.5%)	(28.9%)	
	High	28	21	16	17	21	25	
	N = 128	(21.9%)	(16.4%)	(12.5%)	(13.3%)	(16.4%)	(19.5%)	
Milk (including flavored milk, cocoa, coffee on milk)	Low	53	12	15	16	7	3	0.12
	N = 106	(50.0%)	(11.3%)	(14.2%)	(15.1%)	(6.6%)	(2.8%)	
	Averages	43	8	5	5	12	3	
	N = 76	(56.6%)	(10.5%)	(6.6%)	(6.6%)	(15.8%)	(3.9%)	
	High	61	20	11	11	15	10	
	N = 128	(47.7%)	(15.6%)	(8.6%)	(8.6%)	(11.7%)	(7.8%)	
Natural fermented dairy drinks, e.g., yogurt, kefir	Low	27	18	18	27	14	2	0.12
	N = 106	(25.5%)	(17.0%)	(17.0%)	(25.5%)	(13.2%)	(1.9%)	
	Averages	19	15	10	16	11	5	
	N = 76	(25.0%)	(19.7%)	(13.2%)	(21.1%)	(14.5%)	(6.6%)	
	High	47	21	23	13	20	4	
	N = 128	(36.7%)	(16.4%)	(18.0%)	(10.2%)	(15.6%)	(3.1%)	
Flavored fermented dairy drinks, e.g., yogurts. kefirs	Low	54	15	14	12	10	1	0.48
	N = 106	(50.9%)	(14.2%)	(13.2%)	(11.3%)	(9.4%)	(0.9%)	
	Averages	29	15	6	12	9	5	
	N = 76	(38.2%)	(19.7%)	(7.9%)	(15.8%)	(11.8%)	(6.6%)	
	High	56	17	16	17	15	7	
	N = 128	(43.8%)	(13.3%)	(12.5%)	(13.3%)	(11.7%)	(5.5%)	
Natural cottage cheese (including natural homogenized cheese)	Low	33	21	17	27	8	0	0.08
	N = 106	(31.1%)	(19.8%)	(16.0%)	(25.5%)	(7.5%)	(0.0%)	
	Averages	34	12	7	11	9	3	
	N = 76	(44.7%)	(15.8%)	(9.2%)	(14.5%)	(11.8%)	(3.9%)	
	High	59	26	14	17	9	3	
	N = 128	(46.1%)	(20.3%)	(10.9%)	(13.3%)	(7.0%)	(2.3%)	
Flavored cottage cheese including homogenized cheese, cottage cheese desserts)	Low	54	24	12	11	5	0	0.01
	N = 106	(50.9%)	(22.6%)	(11.3%)	(10.4%)	(4.7%)	(0.0%)	
	Averages	45	12	4	3	8	4	
	N = 76	(59.2%)	(15.8%)	(5.3%)	(3.9%)	(10.5%)	(5.3%)	
	High	59	19	21	18	9	2	
	N = 128	(46.1%)	(14.8%)	(16.4%)	(14.1%)	(7.0%)	(1.6%)	
Yellow cheeses (including processed cheese, blue cheese)	Low	43	21	14	20	7	1	0.55
	N = 106	(40.6%)	(19.8%)	(13.2%)	(18.9%)	(6.6%)	(0.9%)	
	Averages	42	11	10	7	4	2	
	N = 76	(55.3%)	(14.5%)	(13.2%)	(9.2%)	(5.3%)	(2.6%)	
	High	70	18	13	17	8	2	
	N = 128	(54.7%)	(14.1%)	(10.2%)	(13.3%)	(6.3%)	(1.6%)	
Sausages. sausages or wieners	Low	16	15	24	26	20	5	0.64
	N = 106	(15.1%)	(14.2%)	(22.6%)	(24.5%)	(18.9%)	(4.7%)	
	Averages	19	12	11	22	11	1	
	N = 76	(25.0%)	(15.8%)	(14.5%)	(28.9%)	(14.5%)	(1.3%)	
	High	22	16	22	36	25	7	
	N = 128	(17.2%)	(12.5%)	(17.2%)	(28.1%)	(19.5%)	(5.5%)	
Dishes made from so-called red meat, e.g., pork, beef, veal, mutton	Low	11	38	23	27	7	0	0.03
	N = 106	(10.4%)	(35.8%)	(21.7%)	(25.5%)	(6.6%)	(0.0%)	
	Averages	12	16	21	22	4	1	
	N = 76	(15.8%)	(21.1%)	(27.6%)	(28.9%)	(5.3%)	(1.3%)	
	High	37	29	28	25	8	1	
	N = 128	(28.9%)	(22.7%)	(21.9%)	(19.5%)	(6.3%)	(0.8%)	
Dishes made from so-called white meat, e.g., chicken, turkey, rabbit	Low	2	8	25	53	16	2	0.56
	N = 106	(1.9%)	(7.5%)	(23.6%)	(50.0%)	(15.1%)	(1.9%)	
	Averages	3	6	21	37	8	1	
	N = 76	(3.9%)	(7.9%)	(27.6%)	(48.7%)	(10.5%)	(1.3%)	
	High	2	19	23	65	15	4	
	N = 128	(1.6%)	(14.8%)	(18.0%)	(50.8%)	(11.7%)	(3.1%)	
Fish	Low	3	30	44	23	6	0	0.00
	N = 106	(2.8%)	(28.3%)	(41.5%)	(21.7%)	(5.7%)	(0.0%)	
	Averages	4	14	34	21	2	1	
	N = 76	(5.3%)	(18.4%)	(44.7%)	(27.6%)	(2.6%)	(1.3%)	
	High	21	45	41	19	1	1	
	N = 128	(16.4%)	(35.2%)	(32.0%)	(14.8%)	(0.8%)	(0.8%)	
Legume seed dishes, e.g., beans, peas, soybeans, lentils	Low	19	40	28	12	6	1	0.00
	N = 106	(17.9%)	(37.7%)	(26.4%)	(11.3%)	(5.7%)	(0.9%)	
	Averages	17	24	19	15	1	0	
	N = 76	(22.4%)	(31.6%)	(25.0%)	(19.7%)	(1.3%)	(0.0%)	
	High	48	50	16	11	3	0	
	N = 128	(37.5%)	(39.1%)	(12.5%)	(8.6%)	(2.3%)	(0.0%)	
Fruits	Low	0	7	8	19	32	40	0.0547
	N = 106	(0.0%)	(6.6%)	(7.5%)	(17.9%)	(30.2%)	(37.7%)	
	Averages	2	4	11	12	27	20	
	N = 76	(2.6%)	(5.3%)	(14.5%)	(15.8%)	(35.5%)	(26.3%)	
	High	5	16	14	29	38	26	
	N = 128	(3.9%)	(12.5%)	(10.9%)	(22.7%)	(29.7%)	(20.3%)	
Vegetables	Low	0	4	10	15	26	51	0.00
	N = 106	(0.0%)	(3.8%)	(9.4%)	(14.2%)	(24.5%)	(48.1%)	
	Averages	2	4	5	18	26	21	
	N = 76	(2.6%)	(5.3%)	(6.6%)	(23.7%)	(34.2%)	(27.6%)	
	High	6	16	16	25	42	23	
	N = 128	(4.7%)	(12.5%)	(12.5%)	(19.5%)	(32.8%)	(18.0%)	
Sweets. e.g., candies, cookies, cakes, chocolate bars, ‘muesli’ bars.	Low	37	25	17	12	10	5	0.53
	N = 106	(34.9%)	(23.6%)	(16.0%)	(11.3%)	(9.4%)	(4.7%)	
	Averages	25	17	10	16	7	1	
	N = 76	(32.9%)	(22.4%)	(13.2%)	(21.1%)	(9.2%)	(1.3%)	
	High	39	37	14	26	10	2	
	N = 128	(30.5%)	(28.9%)	(10.9%)	(20.3%)	(7.8%)	(1.6%)	
Nuts	Low	36	30	18	10	10	2	0.00
	N = 106	(34.0%)	(28.3%)	(17.0%)	(9.4%)	(9.4%)	(1.9%)	
	Averages	31	15	16	10	4	0	
	N = 76	(40.8%)	(19.7%)	(21.1%)	(13.2%)	(5.3%)	(0.0%)	
	High	80	18	13	11	5	1	
	N = 128	(62.5%)	(14.1%)	(10.2%)	(8.6%)	(3.9%)	(0.8%)	
Powdered or ready-made soups, e.g., from a can. jar	Low	89	12	3	1	1	0	0.63
	N = 106	(84.0%)	(11.3%)	(2.8%)	(0.9%)	(0.9%)	(0.0%)	
	Averages	61	7	3	3	1	1	
	N = 76	(80.3%)	(9.2%)	(3.9%)	(3.9%)	(1.3%)	(1.3%)	
	High	104	16	4	1	3	0	
	N = 128	(81.3%)	(12.5%)	(3.1%)	(0.8%)	(2.3%)	(0.0%)	
Preserved. pickled products	Low	64	30	6	5	1	0	0.07
	N = 106	(60.4%)	(28.3%)	(5.7%)	(4.7%)	(0.9%)	(0.0%)	
	Averages	52	14	6	3	1	0	
	N = 76	(68.4%)	(18.4%)	(7.9%)	(3.9%)	(1.3%)	(0.0%)	
	High	103	18	5	1	0	1	
	N = 128	(80.5%)	(14.1%)	(3.9%)	(0.8%)	(0.0%)	(0.8%)	

## Data Availability

The data presented in this study are available on request from the corresponding author due to privacy.
